# A Therapeutic Approach Using the Combined Application of Virtual Reality with Robotics for the Treatment of Patients with Spinal Cord Injury: A Systematic Review

**DOI:** 10.3390/ijerph19148772

**Published:** 2022-07-19

**Authors:** Amaranta De Miguel-Rubio, Lorena Muñoz-Pérez, Alvaro Alba-Rueda, Mariana Arias-Avila, Daiana Priscila Rodrigues-de-Souza

**Affiliations:** 1 Department of Nursing, Pharmacology and Physiotherapy, University of Cordoba, 14004 Cordoba, Spain; t62mupel@uco.es (L.M.-P.); b42alrua@uco.es (A.A.-R.); 2 Physical Therapy Department, Universidade Federal de São Carlos, São Carlos, São Paulo 13565-905, Brazil; m.avila@ufscar.br; 3 Physiotherapy Section, Faculty of Medicine and Nursing, University of Cordoba, 14004 Cordoba, Spain; drodrigues@uco.es

**Keywords:** spinal cord injury, virtual reality, robotic devices, brain–machine interface, physical therapy, systematic review, rehabilitation

## Abstract

Spinal cord injury (SCI) has been associated with high mortality rates. Thanks to the multidisciplinary vision and approach of SCI, including the application of new technologies in the field of neurorehabilitation, people with SCI can survive and prosper after injury. The main aim of this systematic review was to analyze the effectiveness of the combined use of VR and robotics in the treatment of patients with SCI. The literature search was performed between May and July 2021 in the Cochrane Central Register of Controlled Trials, Physiotherapy Evidence Database (PEDro), PubMed, and Web of Science. The methodological quality of each study was assessed using the SCIRE system and the PEDro scale, whereas the risk of bias was analyzed using the Cochrane Collaboration’s tool. A total of six studies, involving 63 participants, were included in this systematic review. Relevant changes were found in the upper limbs, with improvements of shoulder and upper arm mobility, as well as the strengthening of weaker muscles. Combined rehabilitation may be a valuable approach to improve motor function in SCI patients. Nonetheless, further research is necessary, with a larger patient sample and a longer duration.

## 1. Introduction

Since 2007, the incidence of spinal cord injury (SCI) in Europe has been increasing, with 2.3 cases of traumatic SCI recorded worldwide per hundred thousand inhabitants [1]. In Spain, the percentage is 12–20 cases per million inhabitants per year [2], the majority of which (81.5%) are SCI of traumatic origin. Of these, 52.4% are due to motor vehicle accidents, followed by falls from height and suicide attempts [3,4,5]. In Spain, the prevalence of traumatic SCI in relation to gender is 4:1 (men: women); however, in the case of non-traumatic SCI, the relationship is 1:1 [6]. The average age at the time of injury is between 30 and 40 years; although two peaks of incidence exist, between the ages of 20–29 years, and 60–69 years [2]. Regarding the level of injury, 50% of cases involve the cervical level, mostly affecting the C5 vertebra followed by: C6, D12, C7 and L1 [7].

Spinal cord injury (SCI) has been historically associated with high mortality rates; however, today it can be seen as a personal and societal challenge [8]. When there is a major disability following a central nervous system (CNS) injury, such as that resulting from an SCI, all areas of the individual’s personal life are affected, as well as those of the family members [9]. Temporary or permanent alterations in sensitivity, mobility (specifically, loss of muscle control), or autonomic function below the level of injury are consequences of the interruption of sensorimotor signals conducted by the spinal cord. Consequently, the ability to perform activities of daily living (ADL) is impaired, which has a negative impact on quality of life [10,11].

The ASIA scale (American Spinal Injury Association) measures the preservation of motor and sensory functions in people with SCI. Injuries are classified into five grades based on the absence of preservation of these functions. Grade A corresponds to complete injury, followed by grades B and C, indicating incomplete injury, and, finally, in smaller proportions, grades D, incomplete injury and E where there is no involvement [12].

Thanks to the improved treatment offered by healthcare professionals, and to a multidisciplinary approach, affected individuals can survive, live, and thrive after sustaining this injury [8]. The ultimate objective of physiotherapy in this type of injury is to ensure that the patient with SCI achieves social reintegration as soon as possible, with regular follow-ups, and plays an active role in both the treatment and the prevention of complications derived from their pathology [9].

One of the cornerstones of neuroscience is neuroplasticity or regenerative capacity, a key characteristic of the central nervous system involved in development and maturity [13]. Task repetition is considered essential for the establishment of movement patterns [14]. With targeted, task-directed training, neural circuits are activated which are responsible for eliciting motor patterns, producing a benefit in sensory functions, and regulating afferent input that mimic the ADL task [15]. Hence, health professionals recommend people with SCI to practice physical activity, since it is reported that only 20–50% are physically active [16]. The possible interventions include stretching, postural treatment, kinesitherapy, hydrotherapy and electrostimulation [17].

In addition, rehabilitation is enhanced when patients are encouraged to participate actively in their treatment, especially in environments that are motivating [18]. These are important aspects which are addressed by novel technologies applied to the field of neurorehabilitation, such as robotic devices, brain-machine interface systems, and virtual reality (VR) [5]. Likewise, it has been proven that neurons accelerate their activity when the adult individual observes the movements performed by another person. Therefore, VR can activate the mirror neuron system, which enhances cortical reorganization and, consequently, functional recovery [19]. The use of VR has increased in recent years, and currently VR is fast becoming a therapeutic option for rehabilitation in neurological disorders. Two types of VR systems exist, according to the level of immersion: semi-immersive or non-immersive systems, and immersive systems. Semi-immersive and non-immersive systems use a screen to display the environment with a low level of immersion. Commercial videogame consoles are included in this type of VR. Immersive systems offer full integration of the user into the virtual environment and these systems can incorporate other devices (e.g., gloves, exoskeletons, etc.) to provide sensory inputs to the patient. Examples of immersive VR systems include VR caves, large-screen projections, and head-mounted displays [20]. The use of VR combined with telemedicine could be a promising approach in the rehabilitation of motor impairment as a consequence of neurological disorders [21]. Additionally, in many cases, VR is incorporated into robotic devices as a complementary and motivating element such as the VR module that accompanies the most recent versions of Lokomat (Hocoma AG; Volketswil, Switzerland) [22]. In the last 15 years, these systems have undergone great development, both for their potential in terms of treatment efficacy and cost-effectiveness, and for offering therapies based on high-intensity repetition [23]. Most of these systems have been investigated in patients with stroke [24,25,26,27], cerebral palsy [28,29], Parkinson’s disease [30,31], and multiple sclerosis [32,33,34].

Therefore, this systematic review aimed to evaluate the effectiveness of the combined use of VR and robotics in the treatment of patients with SCI.

## 2. Materials and Methods

### 2.1. Literature Search

This review was performed according to the PRISMA (preferred reporting items for systematic reviews and meta-analyses) guidelines [35]. A complete checklist, according to the PRISMA statement, is reported in Appendix A.

A search of the following scientific literature databases was conducted: PubMed, Web of Science, PEDro and Cochrane Central Register for Controlled Trials, including articles published from January 2011 to July 2021. The electronic search strategy included the following keywords: (“spinal cord injury” OR “spinal cord injuries” [MeSH] OR “paraplegia” [MeSH]) AND (“virtual reality” [MeSH] OR “virtual reality exposure therapy” [MeSH] OR “video game”) AND (“robotics” [MeSH] OR “exoskeleton device” [MeSH] OR “neurorobotics” OR “lokomat”). In addition to the searches in databases and electronic journals, we also examined the bibliographic reference sections of the articles selected for this review, to be added if they met the inclusion criteria.

### 2.2. Selection Criteria

The PICOS (population, intervention, comparison, outcomes and study design) model was employed to define the selection criteria, where the population was adults diagnosed with SCI; the intervention was the combined use of VR and robotics in the treatment of patients with SCI; comparison was adults with and without SCI who performed both combined VR and CPT; the outcomes were related to mobility and functionality; and the study design included one case report, case series and clinical assays as randomized-controlled trials (RCTs) and nonrandomized trials. The following exclusion criteria were considered: studies in which participants were people with SCI and other pathologies, for which the outcome data were not provided for each specific population. Furthermore, publications without available full-text manuscripts and in the form of abstracts and reviews were excluded. The remaining articles were rigorously analyzed to obtain the articles included in the systematic review. Two reviewers (A.M.R. and L.M.P.) took part independently in the study selection process, review, and systematic data extraction. A third reviewer (D.P.R.S.) participated in the final decision in cases of doubt.

### 2.3. Data Extraction

The following data were extracted from each article: author, country, number of participants, age of sample, sex of sample, ASIA grade, level of injury, time since injury, type of study, level of evidence, type of intervention, intensity of session, duration of session, duration of intervention, variables studied, measurement instruments and results obtained.

### 2.4. Quality Assessment

The Cochrane Collaboration’s tool [36] was used to analyze the risk of bias, developed by the Review Manager 5.3 software (Copenhagen, Denmark). This tool includes an evaluation of different items in terms of risk of bias. The studies are categorized as: “unclear risk”, “low risk”, and “high risk”. Two reviewers conducted the risk of bias assessment. In cases of doubt, a third assessor took part in the final decision.

The methodological quality of each study was assessed using the Spinal Cord Injury Rehabilitation Evidence (SCIRE) system [37] and the Physiotherapy Evidence Database (PEDro) scale [38]. Using SCIRE and PEDro, the level of evidence of each of the selected studies was classified. The combination of these two systems (SCIRE-PEDro), uses different categories to analyze the research design and methodological quality, grading from level 1 (highest quality) to 5 (lowest quality). For the classification of RCTs according to levels of evidence 1 or 2, the PEDro scale was used. This scale comprises different items related to the domains of selection, performance, detection, information, and attribution bases. A higher score shows a higher methodological quality. Studies with PEDro scores of six or higher are considered of high methodological quality (6–8: good; 9–10: excellent), and studies with scores of five or less are considered of low methodological quality (4–5: acceptable; <4: poor) [39]

### 2.5. Data Synthesis

A systematic review (qualitative synthesis) was performed, since the variables studied and the type of treatment of the randomized controlled trials included were heterogeneous, and therefore, a meta-analysis (quantitative synthesis) could not be performed.

## 3. Results

**Figure 1:**
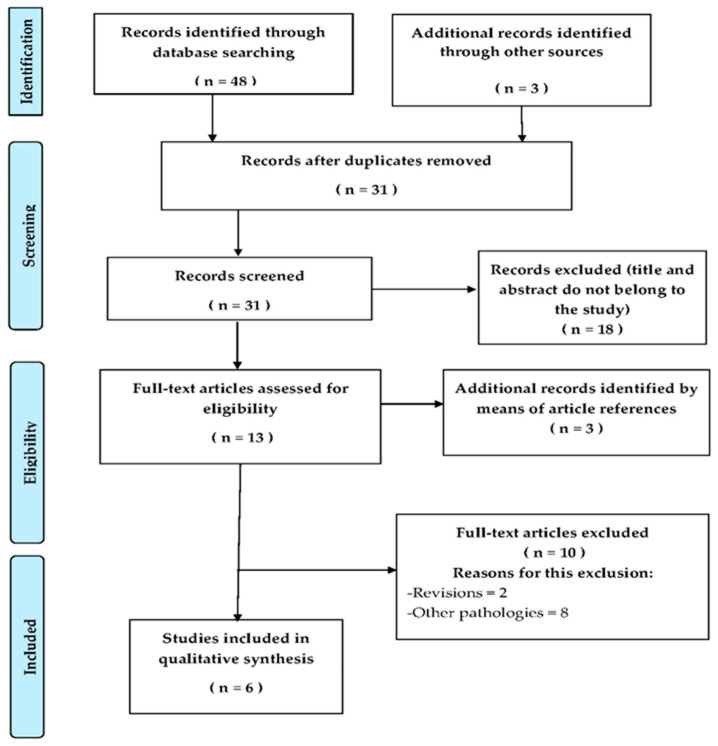
Flow diagram of the selection process of the systematic review following the PRISMA recommendations.

The selection process of this systematic review is shown in Figure 1, retrieving a total of 48 potentially relevant articles: two in PubMed, four in Cochrane Central Register for Controlled Trials, 18 in PEDro and 24 in Web of Science. A total of six studies were included in this systematic review.

The six selected articles were those by Calabrò et al. [40], Casadio et al. [41], Dimbwadyo-Terrer et al. [42], Kowalczewski et al. [43], Prochazka and Kowalczewski [44] and Tidoni et al. [45].

### 3.1. Summary of the Main Results

Of the total sample size (*n* = 63), the study samples ranged from a minimum of 9 and a maximum of 14 participants per study, with the exception of the single participant featured in the case report by Calabrò et al. [40]. In terms of gender, 13 women were included and 37 men, and in one study the sex of the 13 participants was not specified [44]. The mean age was reported in five studies, except for the study by Prochazka and Kowalczewski [44], and the control group (CG) of Casadio et al. [41] which only specified the range, with the youngest subjects in the intervention group (IG) being those of Tidoni et al. [45] and the oldest IG subjects being those of Dimbwadyo-Terrer et al. [42]. Overall, 39 participants had complete SCI, six had incomplete SCI, and 18 were healthy subjects, most studies included participants injured at cervical (80%) or thoracic (20%) levels. The main characteristics of the participants are shown in Table 1.

In terms of combination therapies, research has been conducted with Lokomat Pro [40], a body-machine interface (BMI) [41], a CyberTouch data glove [42], brain-computer interface (BCI) [45] and two studies used ReJoyce [43,44]. The type of VR used in combination with these devices was non-immersive in four studies [40,43,44,45], whereas one study used immersive VR [42] and in another study, both types were mixed [41]. Total sessions range from 4 to 40 sessions, divided between 2 and 8 weeks, although one study did not specify the number of weeks [45]. Table 2 shows the main characteristics of the interventions carried out in the different studies.

### 3.2. Asessment of the Risk of Bias and Methodological Quality of the Studies Included in the Review

**Figure 2:**
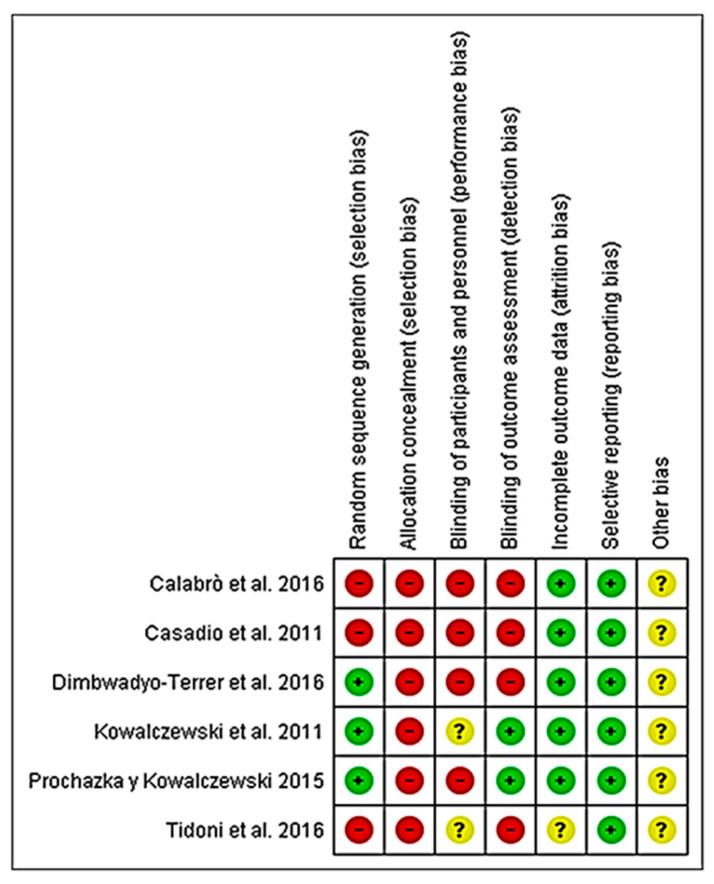
Risk of bias of the studies included in the systematic review [40,41,42,43,44,45].

**Figure 3:**
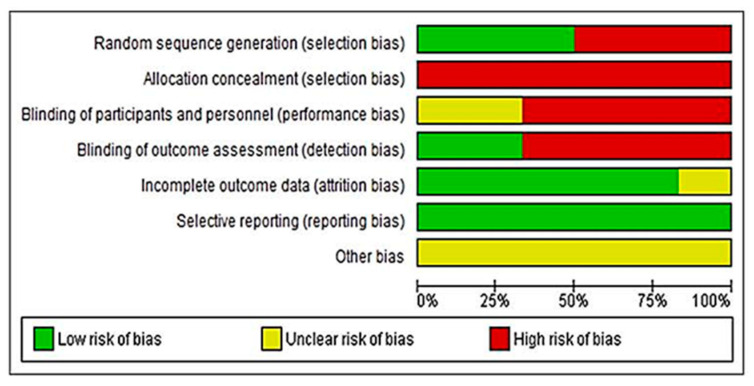
Overall risk of bias. Each category is presented by percentages.

Figure 2 and Figure 3 summarize the risk of bias assessment of the included studies, both globally and individually for each study. When analyzed individually (Figure 2), the study by Kowalczewski et al. [43] has the lowest risk of bias, followed by Prochazka and Kowalczewski [44]. In contrast, the studies by Calabrò et al. [40] and Casadio et al. [41], show the highest risk of bias. Overall, (Figure 3) 100% of the biases appear when selection biases are evaluated.

The validity of the studies included in the present review, estimated by determining the risk of bias among all the studies (Figure 3), shows that the lowest risk of bias is due to partial information of the results (0%), followed by incomplete data (20%). Likewise, when comparing the risk of bias for each of the included studies, overall, randomized controlled clinical trials presented the lowest risk of bias (Figure 2).

The methodological quality of the randomized controlled trials included in this review was generally good (average total PEDro score = 6.3, range 6–7). Three [42,43,44] studies had a high methodological quality, scoring equal to or higher than six points, as shown in Table 3. In addition, the other studies obtained a level four and five of evidence according to the SCIRE-PEDro criteria (Table 2).

## 4. Discussion

The aim of this study was to estimate the effectiveness of a therapeutic approach based on the combined application of robotics with VR in patients with SCI. Six articles were selected for this purpose, of which three were randomized controlled clinical trials [42,43,44], one was a post-test [45], the other was a pre-post-test [41] and the third was a case report [40].

Two articles [41,45] combined interface systems with VR and four of them [40,42,43,44] combined robotic devices with VR. The most relevant outcomes in the upper limbs were: increased residual shoulder mobility [41], significant improvement (11 points in spinal cord independence measure (SCIM) [42], ReJoyce arm and hand function test (RAHFT) data of 1.8 for functionality focused on activities of daily living [44]. Regarding the lower limbs, a decrease in knee and hip joint stiffness and an increase in hip flexion–extension strength were obtained after 40 treatment sessions [40].

According to the International Classification of Functioning, Disability and Health (ICF), we can hypothesize that ULMF (upper limb motor function) impairments influence the loss of functional performance, since impairments at the body structure and functional level can influence activity limitations and participation restrictions [46].

In the study by Calabró et al. [40], two treatments were compared in the same patient with incomplete SCI. Both used Lokomat Pro, a robotic orthosis that mobilizes the lower limbs, assists walking, and includes a screen within its structure that provides feedback in the form of VR. This study was focused on the lower limbs, unlike the other studies consulted, which were more targeted towards rehabilitation of the upper limbs. Although better results were obtained (low level of evidence, *n* = 1) in therapy where the device was combined with rTMS (repetitive transcranial magnetic stimulation), one cannot rule out that this may be due to the placebo effect (by rTMS) and to the previous treatment sessions with Lokomat Pro alone. However, growing evidence in the literature supports the absence of a placebo effect for rTMS [47]. Therefore, the beneficial effects of such research (rTMS + Lokomat Pro) could be reliable due to the neuromodulation properties of the rTMS procedure that was applied. Indeed, it has been shown that rTMS could decrease intracortical inhibition phenomena and form I-waves, enabling the recruitment of preserved corticospinal tract fibers and thus improving motor functions [48]. In particular, the functional recovery induced by rTMS does not appear to depend on the improvement of spinal conductivity, since the latencies of evoked potentials did not change. Therefore, it is possible to hypothesize that rTMS could have induced compensatory plasticity mechanisms and recruited stunned or dysfunctional spinal motor neurons, as suggested by the significant increase in MUNE (motor unit estimation number) [49]. In addition, the rTMS + Lokomat Pro protocol significantly reduced the patient’s lower extremity stiffness, which significantly influences the recovery of motor function. It is worth highlighting that Casadio et al. [41] and Tidoni et al. [45] were the only studies in which the CG was composed of healthy patients, therefore, further attention should be paid to those that include SCI patients in this group. Likewise, these researchers shared the use of interface systems in their studies. Interface systems are defined as communication and/or control systems that allow an interaction between the brain or body and external devices in real time [5]. BCIs analyze brain signals, convert them into real-time output commands that do not depend on common efferent pathways (spinal cord, peripheral nerves, muscles), and transform them into a useful signal to control an external device [50]. In turn, the BMI maps the users’ residual motor skills into efficient control patterns [41].

Reduced or absent mobility of the upper arms and/or hands limits the use of the shoulder in activities of daily living. This contributes to shoulder weakness, poor posture and, over time, produces pain and attenuates voluntary control of shoulder motion [51,52]. In this regard, Casadio et al. [41] obtained statistically significant changes in MMT (manual muscle test) in the IG, which suggests that the training proposed in their study is adequate to exercise all available degrees of freedom in the upper body through the specific practice of controlled actions in VR environments, and consequently, this would facilitate the strengthening of the weaker musculature. This initial study supports the feasibility of using the same controller to solve tasks with different operational functions. According to Kantak, et al. [53], training based on different tasks has a beneficial effect on the learning process, because it induces a broader knowledge of the possibilities offered by the controller and requires a more versatile reorganization of body movements. The balance between being able to perform exercise involving underused muscles in these people and the ease of controlling this device prevents atrophy and improves the recovery process [41].

Tidoni et al. [45] obtained a variability of results in the three IG patients in relation to the CG. In addition, it is important to note that patient 2 only participated in one part of the treatment, i.e., VR, without receiving robotic therapy. No significant differences were found in the variables studied between the two groups; however, it is important to mention that the subjective experience of the subjects with SCI did not differ from the healthy subjects. These results extend previous findings that found improvements in control using a motor imagery-based BCI when proprioceptive stimulation was combined with visual feedback in a group of healthy subjects [54,55,56].

Another issue worth noting is that the study by Dimbwadyo-Terrer et al. [42] applied conventional physical therapy (CPT) together with VR. Realistically, this would hamper the observation of favorable results due to VR because of the longer IG training time. A study by Dimbwadyo-Terrer et al. [42] provided vibrotactile feedback to the hand using the CyberTouch glove, resulting in a tendency towards improved MB (muscle balance) values and changes in functional and clinical parameters in the IG. This suggests that, thanks to the gain in muscle strength of the muscle groups involved in a given movement, better functionality and precision is achieved. This idea was shared by Casadio et al. [41], who stated that muscle strengthening plays a major role in patient improvement. However, it is not possible to attribute this achievement exclusively to new technologies since CPT was applied in parallel. In contrast to Dimbwadyo-Terrer et al. [42], Kowalczewski et al. [43] allowed 1 month of rest between CPT and VR robot treatment, which allowed the effects produced by the two therapies to be independently differentiated. In this case, the results on the ARAT (action research arm test) and RAHFT indicated that the group using ReJoyce displayed statistically significant and clinically important improvements over CPT. The intensity and repetitive action offered by this device together with the challenge of performing movements that gradually increase in speed and complexity make its effectiveness remarkable.

Two studies [43,44] used the same type of robot, ReJoyce, also using the longest intervention time, 60 min. This system consists of an articulated and segmented robotic arm that allows a wide variety of movements of the upper limbs: right and left, up and down, in and out, grip, turning a doorknob or a key. In addition, it has a computer screen on which the various VR games are played, providing feedback to the participating subject. In the study by Kowalczewski et al. [43] the same treatment was applied to both the CG and IG, although in a different order. Prochazka and Kowalczewski [44] evaluated the function in the ADLs and the range of motion (ROM) of each upper limb joint. The data for the RAHFT was 1.8, therefore, bearing in mind that above 0.8 is considered a great change, its effect has validity in functional recovery directed to ADL. It is worth mentioning that the participants had good to very good ROM in the elbow and shoulder at baseline, and therefore, this could have helped to obtain this result. It would be interesting to apply scales such as the SCIM used by Dimbwadyo-Terrer et al. [42], to determine in which areas more independence is gained. Both studies [43,44] demonstrate that it is possible to receive VR treatment at the patient’s home while a physiotherapist supervises the therapeutic exercise, provided that the patient has an Internet connection. The favorable results led to 9 out of 13 participants repeating the process with the other hand in the first study [43] and 5 out of 13 repeating the process in the second study [44]. One aspect that remains to be demonstrated is the validity of the absence of constant supervision by the therapist for patients to do daily treatment from home at any time of the day [44].

Although this study shows some relevant findings, certain limitations should be considered. It is important to highlight that the study search revealed few papers that applied VR together with robotics in patients with SCI. Therefore, these results should be taken with caution. One limitation of the study is that the search strategy carried out did not include only MeSH terms and excluded grey literature publications. The small sample of participants, the short duration of the treatment and the variety of measuring instruments in each of the studies should also be noted as difficulties in making a meta-analysis for significant pooled results. In addition, the levels of injury differed and, consequently, so did the results obtained; in most cases, complete spinal cord injuries were reported, and therefore no major changes in functional terms could be expected in these participants. Some studies included healthy subjects, which, together with the aforementioned limitations, contributes to the scarcity of statistically significant results.

Nonetheless, precisely the fact that a small number of articles were obtained in line with the aim of the present systematic review could be considered a strength of this work, since, in our opinion, it may be a pioneer of this field of research.

## 5. Conclusions

This systematic review sought to identify the effectiveness of immersive and non-immersive VR systems, together with robotics, in patients with SCI. Several different VR systems were used in the studies under review. The only study that includes the two main features within the same device is Lokomat Pro, whereas the other combinations are independent, i.e., they are not part of the same device. The statistically significant changes were found in the upper limbs, where improvements were found in the mobility of the shoulder and upper arms, as well as a strengthening effect on the weaker muscles. Nevertheless, further research is needed to evaluate the functional benefits of this therapy, and to study different protocols in a larger sample of patients, over a greater number of treatment sessions, and focused on a more specific type of patient. It would be useful to train the people who perform the tests to improve application, establish protocols aimed at each type of patient and study what type of exercises are most effective. Undoubtedly, combining science with technology is a promising goal for the future of neurorehabilitation, as it opens a door to further applications of VR as a tool to support physiotherapy interventions.

## Figures and Tables

**Table 1:** Demographic and clinical characteristics of the studies.

Study Country	Participants (*n*)	Age (years) Mean ± SD (Range)	Sex F/M	ASIA Grade	Level of Injury	Time after Onset Injury (Months)
**Calabrò et al., 2016** [40] (Messina) Italy	*n* = 1	31	M	C	Incomplete T10	20
**Casadio et al., 2011** [41] (Chicago) USA	*n* = 14 CG: 8, IG: 6	CG: (21–35) IG: 40.17 ± 3.53	CG: 1F/7M IG: 6M	IG: A (3) C (3)	Complete cervical: C4 (1), C5 (1), C6 (1) Incomplete cervical: C3-C4 (1), C4 (2)	IG: 50.83
**Dimbwadyo-Terreret al., 2016** [42] (Toledo) Spain	*n* = 9 CG:3, IG: 6	CG: 44.17 ± 22.29 IG: 54.3 ± 9.86	CG: 1F/2M IG: 1F/5M	CG: A (3) IG: A (5) D (1)	CG: complete thoracic: T4 (2), T6 (1) IG: complete thoracic: T4 (5) incomplete cervical: C4 (1)	CG: 5 ± 1 IG: 5.83 ± 2.99
**Kowalczewski et al., 2011** [43] (Alberta) Canada	*n* = 13 GC: 7, IG: 6	35.92 ± 11.96	6F/7M	A-B	Complete cervical: C5 (5)-C6 (4)-C7 (4)	3.62 ± 2.12
**Prochazka y Kowalczewski,2015** [44] (Alberta) Canada	*n* = 13. CG: ND IG: ND	(24–56)	ND	A-B	Complete cervical: C5-C6	ND
**Tidoni et al., 2016** [45] (Rome) Italy	*n* = 13 CG: 10, IG: 3	CG: 29.33 ± 2.87 (24–32) IG: 28 ±5.19 (22–31)	CG: 4F/6M IG: 3M	A-B-D	Complete cervical: C4 (1), C4-C5 (1) Incomplete cervical: C6 (1)	IG: 88.67

ASIA: American Spinal Injury Association Impairment Scale; F: female; CG: comparison group; IG: intervention group; M: male; ND: not described.

**Table 2:** Main characteristics of the studies included in the systematic review.

Study SCIRE-PEDro Scores	Group Interventions	Intensity	Session Duration	Intervention Duration	Outcome	Measuring Instrument	Results
**Calabrò et al., 2016** [40] Case report Pre-post test Level 5	IG: Lokomat Pro with motivating feedback in a virtual environment (non-immersive VR) IG (rTMS): Lokomat Pro (non-immersive VR), + repetitive transcranial magnetic stimulation	5 times/week	40 min	8 weeks	Lower limbs: strength and rigidity in flexion/extension of hip and knee.	ASIA, LEMS RMT, MEP CCT, MUNE rigidity, strength, DGF	IG: slight improvement in kinetic parameters (reduces rigidity in knee and hip). No significant changes in clinical or electrophysiological parameters. IG (rTMS): improvement in ASIA (C to D), LEMS (3 to 9) scores, statistically significant reduction of hip and knee stiffness, device guidance force, BWS (61 ± 6% to 57 ± 3%), increase in hip flexion–extension force, MEP amplitude, MUNE, and speed (1.5 ± 0.3 Km/h to 1.7 ± 0.2 Km/h)
**Casadio et al., 2011** [41] Controlled clinical assay. Pre-post test Level 4	CG and IG: VR games, consisting of a virtual board (non immersive VR) and simulated conduction (immersive VR), combined with technologies capturing movement (BMI)	2–3 times/week	45 min	3 weeks	Upper limb: ROM of shoulder. Isometric strength of shoulder in 3 directions.	MMT and normal scale with scoring from 0 to 5 for ROM	MMT improves for all individuals: F (1.5) = 10; *p* = 0.02. Significant correlations between shoulder muscle force in the upper, forward and backward directions and scapular elevation, shoulder protraction and retraction (R = 0.55 *p* = 0.0073, R = 0.72 *p* = 0.0012, R = 0.75 *p* < 0.0001, respectively). Five out of six subjects improved total isometric force.
**Dimbwadyo-Terrer et al., 2016** [42] Clinical assay randomized pilot study. Level 1	CG: CTP IG: immersive VR system + CyberTouch glove. Two session types: one of reaching and throwing movements, and the other only reaching ones. One type per day was performed. Same CTP as CG.	2 times/week	30 min	2 weeks	Upperlimb: motor (muscle strength and self-management, co-ordination and fine motor control.	Functional state: MB, BI, SCIM. NHPT and JHFT scales. Time taken to complete the items.	No significant differences were found in the outcomes between groups, although MB was higher IG. The SCIM scale improved in both groups: >11 points in IG, and >4 points in SCIM self-care for IG (improved skills, coordination and fine movements of the fingers) IG needed shorter time for NHPT.
**Kowalczewski et al., 2011** [43] Randomized controlled clinical assay. Level 1	CG: CPT+ 1 month’s rest + ReJoyce with video games (non immersive VR) that mimic the ADL. IG: ReJoyce with videogames that mimic the ADL. + 1 month’s rest. +CPT.	5 times/week	60 min	6 weeks	Upperlimb: functionality for ADL and ROM.	ARAT RAHFT	IG improved more than CG according to ARAT (13.0% ± 9.8% and 4.0% ± 9.6%, respectively=).
**Prochazka y Kowalczewski 2015** [44] Randomized controlled clinical assay Level 1	CG: telesupervised CPT. IG: FES + sessions telesupervised with ReJoyce (non immersive VR).	6 times/week	60 min	6 weeks	Upperlimb: functionality and ROM. Validity of RAHFT, ARAT and FMA.	RAHFT ARAT FMA	RAHFT is better for studying functionality and FMA for the ROM. Effect sizes of IC group: RAHFT (0.64 ± 3.6) ARAT (1.3 ± 6.3), FMA (1.5 ± 5.2)
**Tidoni et al., 2016** [45] Post-test Level 4	CG and IG: immersive VR of mathematical game with board and proprioceptive stimulator in the brachial biceps tendon with feedback from a video recorded with a robot.	12 times/ND	6 min	ND	Results of questionnaire on user’s experience, optimization calls, and information transfer rate.	UE OC ITR	Patient 1: lesser precision in the task than CG and higher OC and lower ITR (*p* < 0.022). Patient 2: only VR. UE, OC and ITR did not differ from the CG. Patient 3: did not differ from the CG in the robot scenario, although UE and ITR obtained lower scores in VR.

ARAT: action research arm test; ASIA: American Spinal Injury Association impairment scale; ADL: activities of daily living; BI: Barthel Index; BMI: body machine interface; CCT: motor central conduction time; CG: comparison group; CPT: conventional physical therapy; DGF: device guidance force; FES: functional electrical stimulation; FMA: Fugl-Meyer assessment; IG: intervention group; ITR: information transfer rate; JHFT: Jebsen Taylor hand function; LEMS: lower extremity motor score; MB: muscle balance; MEP: motor evoked potential; MMT: manual muscle test; MUNE: motor unit estimation number; ND: not described; NHPT: nine hole peg test; OC: optimization calls; RAHFT: ReJoyce automated hand function test; RMT: resting motor threshold; ROM: range of motion; rTMS: repetitive transcranial magnetic stimulation; VR: virtual reality; SCIM: spinal cord independence measure; UE: user experience.

**Table 3:** PEDro scores obtained by the different studies included in the systematic review.

Study	1	2	3	4	5	6	7	8	9	10	11	Total
**Dimbwadyo-Terrer et al., 2016** [42]	-	YES	NO	YES	NO	NO	NO	YES	YES	YES	YES	6
**Kowalczewski et al., 2011** [43]	-	YES	NO	YES	NO	YES	NO	YES	YES	YES	YES	7
**Prochazka y Kowalczewski, 2015** [44]	-	YES	NO	YES	NO	NO	YES	NO	YES	YES	YES	6

Range: 0–10. Item 1 is not used in the method score. Note: “YES” indicates that a study meets that particular criterion. “NO” means that this study does not meet the criteria or that it does not provide enough information to be sure. 1. Eligibility criteria were specified; 2. Subjects were randomly allocated to groups (in a crossover study, subjects were randomly allocated an order in which treatments were received); 3. Allocation was concealed; 4. The groups were similar at baseline regarding the most important prognostic indicators; 5. There was blinding of all subjects; 6. There was blinding of all therapists who administered the therapy; 7. There was blinding of all assessors who measured at least one key outcome; 8. Measures of at least one key outcome were obtained from more than 85% of the subjects initially allocated to groups; 9. All subjects for whom outcome measures were available received the treatment or control condition as allocated or, where this was not the case, data for at least one key outcome was analyzed by “intention to treat”; 10. The results of between-group statistical comparisons are reported for at least one key outcome; 11. The study provides both point measures and measures of variability for at least one key outcome.

**Figure A1:**
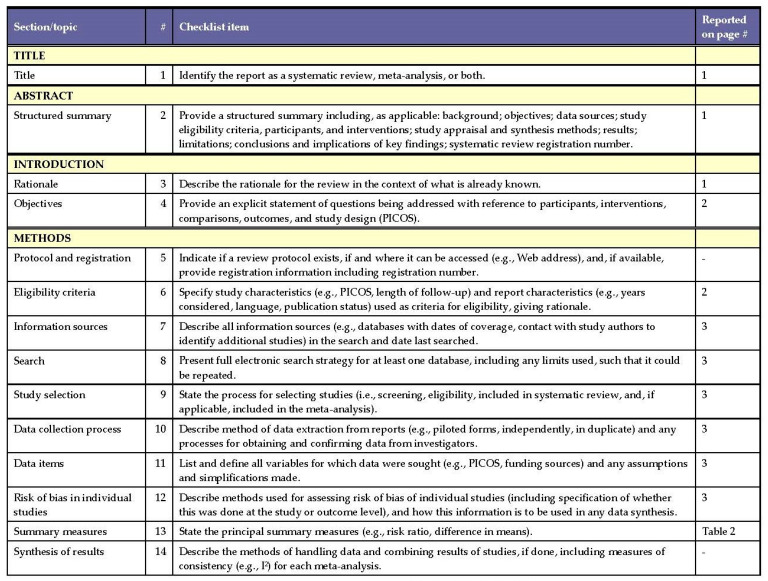
PRISMA Checklist [35].

**Figure A2:**
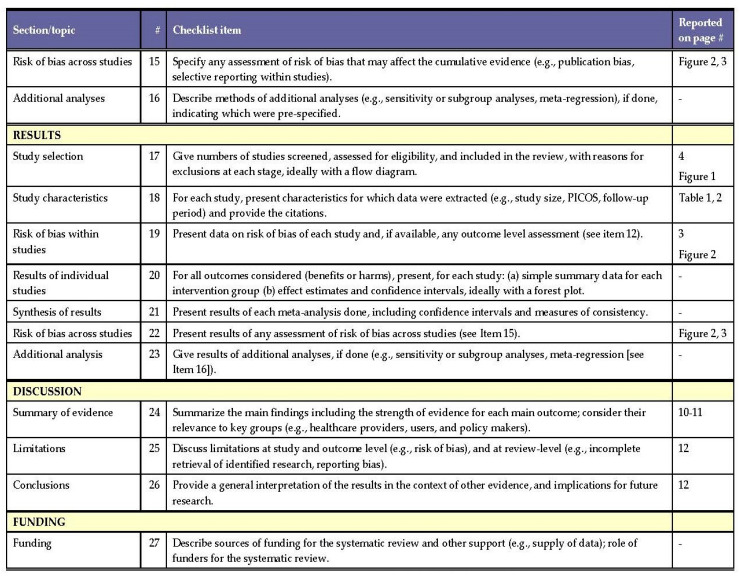
PRISMA Checklist [35].
